# Magnetite (Fe_3_O_4_) Supported on Bagasse Sugarcane Fibers as Catalyst for Plasma-Degradation of Organic Pollutant in Water: Effect of Oxidation Inhibitor Agents on the Particles’ Shape and Catalytic Activity

**DOI:** 10.3390/polym18141730

**Published:** 2026-07-14

**Authors:** Néhémie Miloh, Franck W. Boyom-Tatchemo, Fabrice Nganbe-Ndjock, Albert B. Mbouopda-Poupi, Elie Acayanka, Georges Kamgang-Youbi

**Affiliations:** Faculty of Science, Department of Inorganic Chemistry, University of Yaounde I, Yaounde P.O. Box 812, Cameroon; miloh02071998@gmail.com (N.M.); nganbef@gmail.com (F.N.-N.); poupialbert009@gmail.com (A.B.M.-P.); drkamgangyoubigeorges@gmail.com (G.K.-Y.)

**Keywords:** supported-magnetite, oxidation inhibitor agent, plasma glidarc, bagasse-sugarcane, Fenton catalysis heterogeneous, amaranth red dye

## Abstract

To easily recover and reuse nano-magnetite (Fe_3_O_4_) during the catalytic process, Fe_3_O_4_ is successfully dispersed by coprecipitation of Fe(II, III) salts on anchoring sites of bagasse-sugarcane fibers generated by gliding-arc plasma. Previously, we explored the effect of ascorbic acid (ASC), hydrochloric acid (HCl) and plasma-activated water (PAW) acting as oxidation inhibitors of Fe(II) solution. Prepared materials were characterized by X-ray diffraction (XRD), Fourier transform infrared spectroscopy (FTIR) and scanning electron microscopy coupled with energy dispersive X-ray spectroscopy (SEM-EDX). The obtained results show that the oxidation inhibitor agent influences the morphology, texture and activity of the synthesized bulk-magnetite, where Fe_3_O_4_-nanorods, Fe_3_O_4_-nanospheres and Fe_3_O_4_-nanosheets were, respectively, obtained with PAW, HCl and ASC. The Fenton-plasmacatalytic treatment of amaranth red dye used as a model pollutant for 30 min revealed degradation rates of 53, 79 and 80%, respectively, for Fe_3_O_4_-ASC, Fe_3_O_4_-HCl, and Fe_3_O_4_-PAW each coupled to plasma. The deposition of nano-magnetite on the plasma-activated bagasse-sugarcane fibers (BM) using PAW as the best oxidation inhibitor agent exhibited characteristic FTIR-absorption bands of -OH, -CH_2_ and Fe-O, attesting the bagasse-sugarcane-Fe_3_O_4_ linkage. The supported magnetite revealed a pollutant degradation rate of 99%, which deeply highlights an activity improvement after Fe_3_O_4_ deposition on plasma-activated bagasse sugarcane. The reusability of supported-Fe_3_O_4_ catalyst revealed a pollutant degradation rate of 95% after the fourth cycle, thus highlighting its easy recovery and catalytic stability (reuse).

## 1. Introduction

Several scientific studies have focused on the effective techniques for decontaminating water polluted by organics and/or inorganics. It appears that due to the inadequacy of conventional wastewater treatment techniques, such as coagulation-flocculation and, to some extent, adsorption [1,2,3,4], researchers have developed effective techniques referred to as advanced oxidation processes (AOPs), such as heterogeneous catalysis, photo- and plasmacatalysis [5,6,7,8,9]. These techniques are based on the production of highly reactive hydroxyl radicals (HO^•^), which have proven to be non-selective and effective for the degradation of recalcitrant organic pollutants [5,6,7,8,9]. Among the various prepared materials used as catalysts, nano-Fe_3_O_4_ (magnetite), as a magnetic material, appears more interesting, knowing that, in addition to its good activity, it could allow for an easy separation and reuse, thus promoting a better catalytic efficiency. However, the synthesis routes of magnetite are crucial to tailor the size, morphology and crystallinity, which impaired the overall catalytic performances. Several approaches were developed, including sol-gel, co-precipitation, hydrothermal and microemulsion methods [10,11]. Among these methods, co-precipitation attracts more attention because of its simplicity and the advantage of obtaining homogeneous materials [12]. Unfortunately, this process frequently requires costly and aggressive commercial acids and solvents as oxidation inhibitor agents of Fe(II) to Fe(III) during the synthesis of the magnetite (Fe_3_O_4_) material. As an alternative, recent works have highlighted the oxidizing and acidifying power of Glidarc-type plasma obtained under atmospheric conditions [13]. The plasma route mainly generates HO^•^ and NO^•^ radicals, which recombine to produce hydrogen peroxide (H_2_O_2_), nitric (HNO_3_) and nitrous (HNO_2_) acids that can be used for the synthesis of magnetic ferrites [14]. Knowing that magnetic nanoparticles during some catalytic processes tend to agglomerate rapidly, it becomes necessary to deposit them on a suitable biomass as support to overcome this problem, thereby expanding and protecting their texture (specific surface area and porosity) as an important property during the heterogeneous catalytic route [15,16]. Therefore, heterogenization of the Fenton catalysis route by depositing the active phase (Fe_3_O_4_) on a support limits the formation of iron hydroxide sludge, allowing an easy recovery and reuse of the catalyst in a wide pH range [17]. Many works reported that the deposition of iron oxide nanoparticles on supports, such as zeolites [18], clays [19] or volcanic ash [20], leads to efficient and easy recyclability of the catalyst. The focus is now on cost-effective and eco-friendly catalytic supports, such as agricultural waste, which has been widely used to support FeO_x_ nanoparticles [21,22,23,24,25]. To assure better deposition, activity and stability of the active phase (Fe_3_O_4_ nanoparticles) on the biomass as support, it becomes necessary to clean and functionalize (activate) the biomass surface properly before the deposition. The gliding arc plasma (plasma glidarc) route, thanks to HO^•^ and NO^•^ species and UV light generated [26,27,28], could induce the functionalization of the biomass surface through the anchoring of the polar functions for a better deposition of Fe_3_O_4_ nanoparticles.

In our previous work, we showed that magnetite (Fe_3_O_4_) particles could be deposited on the plasma-activated hyacinth biomass through a co-precipitation method using plasma-activated water (PAW) as an oxidation inhibitor agent of Fe(II) to Fe(III), and the obtained material was used for Fenton catalytic degradation [27]. However, we did not explore the effect of the oxidation inhibitor agent on the physicochemical properties (structure, morphology and texture) and activity of bulk magnetite coprecipitated before its deposition on an efficient support. In addition to PAW, we used other oxidation inhibitor agents, such as ascorbic acid (ASC) and hydrochloric acid (HCl), to prepare bulk Fe_3_O_4_. The best one following the activity is used for the deposition of Fe_3_O_4_ on the plasma-modified bagasse sugarcane fibers as support. The choice of the bagasse sugarcane fibers as support is motivated by the fact that they are more available locally and efficiently used in the literature [21,22,23,24,25]. The water hyacinth biomass used in the previous study, which, despite no sludge generation and easy recovery, revealed a catalytic activity decrease after the second cycle, and significantly after the third cycle [27]. Therefore, plasma-activated bagasse sugarcane fibers, largely available locally, could be more suitable as support for a better deposition of magnetite particles. In the present study, the plasma glidarc route has a multifunctionality: (i) bagasse sugarcane surface activation, (ii) production of activated water (PAW) as an oxidation inhibitor agent of Fe(II) to Fe(III), thanks to the reactive nitrogenous species (RNS: NO^•^, NO_2_^−^, HONO_2_ etc) and oxygenated species (ROS: HO^•^, H_2_O_2_, ^•^O_2_^−^) and (iii) generation of UV light and H_2_O_2_ (through recombination of HO^•^ species plasma-generated) for Fenton plasmacatalytic degradation of the organic pollutant in aqueous solution [26].

The main objective of this work consisted of evaluating the effect of the oxidation inhibitor agent of Fe(II) to Fe(III) on the physicochemical properties of bulk magnetite obtained via co-precipitation of iron salts (II and III). Secondly, a suitable oxidation inhibitor agent is used for better deposition of magnetite particles on the plasma-activated bagasse sugarcane fibers as support. Therefore, the plasma-assisted Fe_3_O_4_ synthesis through the activated water is utilized as a production oxidation inhibitor agent of Fe(II) to Fe(III), on the one hand, and the bagasse sugarcane fibers as an activation support, on the other hand. All the prepared materials are used as Fenton heterogeneous catalysts coupled to the plasma glidarc route for better degradation of the amaranth red dye as a pollutant molecule model. Knowing that dyes are among the most important categories of emerging pollutants, inducing severe carcinogenic and mutagenic effects for living beings, the environment can be threatened (face huge environmental regulations) after their excessive use and release by various industries such as drugs, cosmetics, textiles, and foods [29,30,31,32,33].

## 2. Materials and Methods

### 2.1. Reagents and Synthesis Protocol

HCl (37%), NaOH (99%), ascorbic acid (ASC), FeCl_2_·4H_2_O (98%) and FeCl_3_·6H_2_O (97%) were purchased from Sigma-Aldrich company (Germany). While activated water (PAW) was produced by the gliding arc plasma (Plasma Glidarc) treatment of distilled water for 30 min (Figure S1), as described in our previous work [27]. However, the process of plasma glidarc operation has been largely described in the literature [19,20,26,27,28]. It operates at ambient conditions and consists of three compartments: (i) An electrical power supply component of a high voltage generator (50 Hz, 9 kV, 1 A in the open circuit). (ii) A gas supply consisting of an air compressor and flowmeter for atmospheric air (N_2_, O_2_ and H_2_O) supply. (iii) The core of the reactor properly designed with a double-walled glass equipped with a cooling system for a simple circulation of water, a nozzle and two aluminium electrodes (length 6 cm) with a diverging profile. When the electrodes are subjected to a suitable potential difference (160 mA, 600 V, 96 W), an electric arc is generated at the minimum electrode gap (2 mm), it is pushed by the gas flow and glides along the electrodes until it is extinguished when it is short-circuited by a new arc, thus forming a large plasma plume. The plasmagenic gas used in the study is air saturated with distilled water from bubbling and injected with a flow rate of 13.33 L.min^−1^. The different bulk materials were prepared by co-precipitation of ferric (FeCl_3_·6H_2_O at 1 mol/L) and ferrous (FeCl_2_·4H_2_O at 0.5 mol/L) ion salts using 50 mL of NaOH solution (1 mol/L) for precipitation [27]. Different oxidation inhibitor agents of Fe(II) to Fe(III), such as HCl (1 mol/L), ascorbic acid (1 mol/L) and plasma-activated water solutions, were used to prevent the conversion of Fe(II) to Fe(III) in aqueous media. Afterwards, the different precipitates obtained were recovered by simple filtration and washed with distilled water, followed by acetone, and dried at ambient temperature for 6 h. The materials obtained using different oxidation inhibitor agents are named: M-PAW (for magnetite obtained with plasma-activated water), M-HCl (for magnetite obtained with hydrochloric acid) and M-ASC (for magnetite obtained with ascorbic acid). The sugarcane bagasse fibers used as a support in this work were collected in the Minlongo locality (Centre region of Cameroon). The sampled bagasse was cut into small pieces, cleaned and washed several times with water until colorless water was obtained. To remove eventual impurities, the bagasse was washed several times with hot water for 6 h. Subsequently, it was dried in sunlight for 6 days, crushed, sieved for particle size between 80 and 200 µm to increase the surface of contact and named B.

The surface activation (modification) of bagasse sugarcane fibers was performed using the plasma glidarc route (Figure S1). It consisted of introducing a mixture of 10 g of bagasse fibers and 430 mL of distilled water into the core of the plasma reactor under magnetic stirring. The plasmagenic gas (moist air) is supplied to the reactor using a compressor connected to a flowmeter set at 600–800 L/h. The mixture is exposed to plasma plume for 60 min, and then recovered, filtered, dried in an oven at 100 °C for 9 h and named BP. The deposition of iron oxides on plasma-activated sugarcane bagasse (support) was carried out following the abovementioned route (co-precipitation) for bulk materials, but by adding the support within the initial mixture. Therefore, 6 g of BP support was introduced in 100 mL of NaOH solution (1 M), 30 mL of FeCl_3_·6H_2_O solution (1 M) and 15 mL of FeCl_2_·4H_2_O solution (1 M) in a ½ ratio, with plasma-activated water as an oxidation inhibitor agent of Fe(II). The synthesis route (coprecipitation) was initiated under magnetic stirring for 30 min, and the obtained precipitate was recovered by simple filtration, washed with distilled water and acetone (to remove the eventual remaining unreacted reagents), dried in an oven at 100 °C for 6 h and named BM.

### 2.2. Characterization Techniques

The structural analysis of all prepared materials was performed by XRD and FTIR analyses. Therefore, to determine the crystalline structure, the X-ray pattern of each material was recorded between 10 and 80° at the rate of 3°/min, using a Siemens D5000 diffractometer (China) equipped with Kα copper radiation (k = 1.33 Å), operating at 40 KV and 30 mA. The JCPDS database was used to identify the crystalline phases. FTIR analysis was carried out to find the chemical functions at the surface of each material. Therefore, the FTIR spectrum was recorded via ATR mode within the wavelength range of 4000–400 cm^−1^ after 32 scans with a resolution of 4 cm^−1^, using a Bruker Equinox IFS55 spectrophotometer (China) equipped with a DTGS detector. The surface morphology approach was investigated through SEM analysis using a Philips XL 30 field emission scanning electron microscope (China) operating at an electron accelerating voltage of 20 KV with an objective using the images produced from the secondary electrons. However, EDX analysis coupled to SEM analysis was used to find the elemental composition at the surface of each material. Otherwise, the magnetic test of prepared materials was performed. The zero-point charges (PH_ZPC_) of bagasse sugarcane fibers (biosorbent) and supported magnetite were determined to obtain a better understanding of the adsorbed molecules on the biosorbent surface as a function of pH solution. This consisted of adding 20 mL of NaCl solution (0.05 M) to several flasks (50 mL) containing distilled water, each having a different initial pH_i_, previously adjusted to between 2 and 10 using HCl (0.1 M) or NaOH (0.1 M) solution. In each flask, 0.05 g of material and all the mixtures were stirred at room temperature for 48 h. Then, the final pH of the supernatants is measured, and the pH_ZPC_ value of the material corresponds to the intersection of the abscissa axis with the curve illustrated by Equation (1).

ΔpH (pH_f_ − pH_i_) = f(pH_i_) (1)

### 2.3. Study of the Degradation of AR Dye by the Heterogeneous Fenton Process in Plasma Medium

The activity and stability of prepared materials were assessed during the plasma Fenton catalytic degradation of amaranth red (AR) dye used as a model pollutant (Figure S2). Firstly, the plasma treatment alone (without catalyst) was performed by introducing 430 mL of AR solution at a defined concentration into the reactor under stirring, and then activating the plasma plume for 60 min. The withdrawn samples at defined times were analyzed by spectrophotometry at 520 nm (maximum absorption wavelength of AR) [34] using an Aqualitic spectrophotometer (China). For plasmacatalytic treatment, a defined catalyst mass and 430 mL of AR solution (10 mg/L) were introduced into the reactor under magnetic stirring. The plasma plume was initiated, and the withdrawn samples at defined times were filtered to recover the catalyst, and then the supernatant was analyzed by a spectrophotometer. For reproducibility purposes, each treatment was carried out twice, and then the average was used to obtain values. The degradation rate of the pollutant was determined following Equation (2), where C_0_ and C_t_ represent, respectively, the initial (t = 0 min) and residual concentrations of the pollutant at a defined time. The plasma glidarc device (Figure S1) used for the activation of water is the same for the degradation of AR by the heterogeneous Fenton process, as described by Lesueur et al. in 1988 [35]. Some key parameters, such as the pollutant’s dose and treatment time, were studied. For the recyclability test, the catalyst was recovered after each cycle via simple filtration, dried and reused for the next cycle.

(2) $$\%\;Degradation=\frac{\left(Co-Ct\right)}{Co}*100$$

The kinetic models 1 and 2 were studied and plotted using Equations (3) and (4) to determine the reaction rates and the experimental parameters influencing the degradation. However, to determine the apparent reaction constants and observe the effect of the concentration of pollutant molecules on the latter, the study of Langmuir–Hinshelwood kinetics was carried out. For this purpose, the concentrations of AR were varied from 10 to 100 mg/L at the optimal conditions of plasma treatment with the M-PAW material (magnetite prepared using plasma-activated water). Equations (5) and (6) illustrate the Langmuir–Hinshelwood kinetics, where K_LH_ represents the adsorption equilibrium constant and K_r_ the reaction rate constant.

(3) $$\ln\left(\frac{C}{Co}\right)=f\left(t\right)$$

(4) $$\left(\frac{1}{C}\right)=f\left(t\right)$$

(5) $$r=-\frac{d\left[AR\right]}{dt}=Kr\frac{KLH\left[AR\right]}{1+KLH\left[AR\right]o}=Kapp\left[AR\right]$$

(6) $$\frac{1}{Kapp}=\frac{1}{Kr*KLH}+\frac{\left[AR\right]}{Kr}$$

## 3. Results and Discussion

### 3.1. Materials Characterization

#### 3.1.1. X-Ray Diffractograms

**Figure 1:**
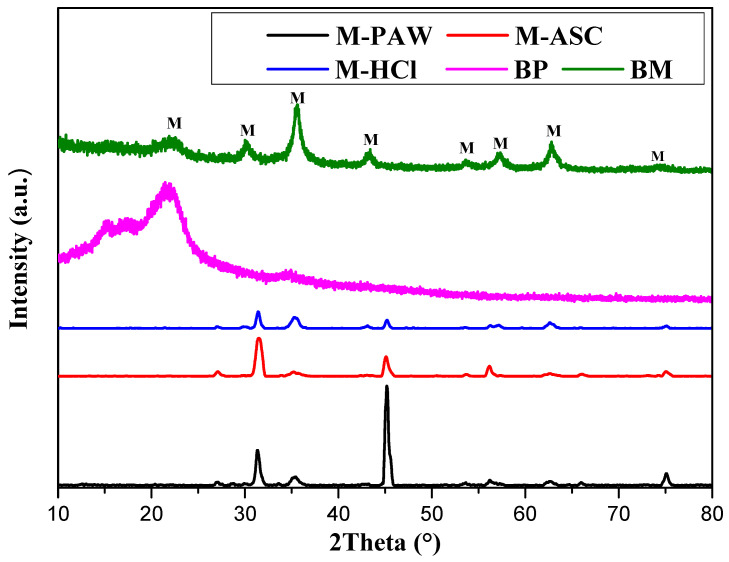
X-ray diffractograms of M-PAW, M-ASC and M-HCl, plasma-activated sugarcane bagasse (BP) and supported magnetite (BM).

The crystalline structure of the prepared bulk materials M-PAW, M-HCl and M-ASC was determined, and the results obtained are depicted in Figure 1. We can see that the three bulk materials have similar crystalline structures, have the same appearance of the peaks position 2ϴ values of 28.14°, 30.4°, 35.1°, 45.2°, 56.6°, 62.6° and 75.1°assigned to the magnetite structure and correspond, respectively, to the diffraction planes (004), (220), (311), (400), (511), (440) and (533) following ICDD 00-019-0629 and corroborated by similar results obtained by Cai and Wan in 2007 [36,37]. The absence of strange peaks could suggest that the prepared bulk materials are pure [38]. Figure 1 also shows the XRD patterns of materials resulting from the plasma activation of the bagasse sugarcane fibers (BP) used as a support and deposited magnetite on the support via the co-precipitation route using plasma-activated water as an oxidation inhibitor agent. However, we can see a dome between 15 and 25° on the plasma-activated bagasse fibers (BP), reflecting the presence of amorphous phases (cellulose). The supported magnetite (BM) pattern shows characteristic peaks at 2ϴ values of 30.9, 36.5, 44.5, 57.7 and 63.5 ascribed, respectively, to the reflection planes of (220), (311), (400), (422) and (511) [39]. The dome hides on the BM pattern, thus highlighting the deposition of magnetite on the biocomposite structure [21].

#### 3.1.2. SEM-EDX and Magnetic Test

**Figure 2:**
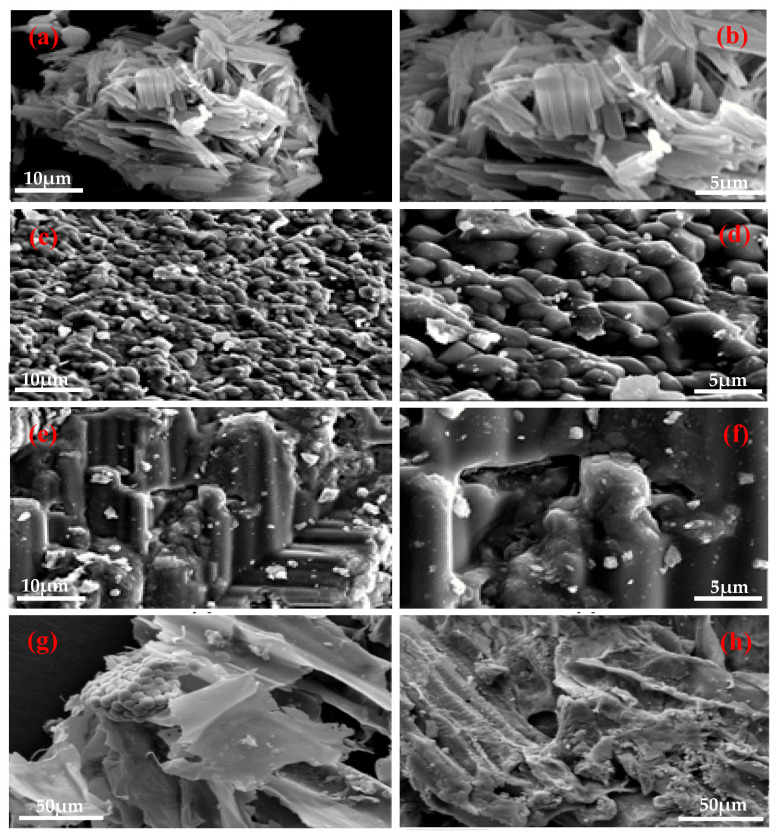
SEM images of M-PAW (**a**,**b**), M-HCl (**c**,**d**) and M-ASC (**e**,**f**), plasma-activated bagasse fibers (**g**) and supported magnetite (**h**).

Figure 2 shows the SEM images of magnetite particles obtained with different antioxidant agents (ASC, HCl, PAW), plasma-modified bagasse fibers and supported magnetite. We can see different topographies depending on the oxidation inhibitor agent used, which reveals that the nature of the antioxidant agent influences the morphology of the particles generated. Therefore, we recorded nanorods (Figure 2a,b), nanospheres (Figure 2c,d) and nanosheets (Figure 2e,f), with heterogeneous particle size distribution, respectively, for magnetite obtained using plasma-activated water, hydrochloric acid and ascorbic acid. The presence of white particles on the materials could be due to the water of crystallization within the structure of magnetite, according to Muhammad et al. in 2022 [38]. The aggregates observed in magnetite prepared using plasma-activated water are due to high humidity [40]. SEM images from Figure 2 also allow a better appreciation of the magnetite particles’ deposition on the plasma-activated bagasse fibers. We can observe that plasma-activated bagasse fibers BP (Figure 2g) have a smooth surface with overlapping and non-compacted sheets with some compacted ball-shaped particles. Supported material BM (Figure 2h) has a rough and compact texture, with magnetite particles on the surface of plasma-activated bagasse fibers, unlike raw bagasse, which is described as without pores according to Toledo-Jardin [41]. EDX spectra of the different materials are shown in Figure S1. The appearance of the sodium element (Na) is attributed to the NaOH reagent used for the co-precipitation of iron salts. Otherwise, the ferromagnetic properties of prepared materials are highlighted through the presence of the iron element in different oxidation states [40]. For plasma-activated sugarcane bagasse (BP) and supported magnetite (BM), we recorded a significant difference in elemental composition. Plasma-activated (BP) and supported magnetite (BM) materials, respectively, revealed 0.78 and 60.38% of iron element, which deeply highlights the deposition of magnetite on the surface of activated bagasse sugarcane fibers.

A magnet test of material prepared with plasma-activated water was carried out, and Figure S3 illustrates the attraction of magnetite particles by a magnet. The materials obtained by co-precipitation of iron salts in the presence of NaOH are perfectly attracted by the magnet, which shows that they are ferromagnetic and are likely to be used in heterogeneous catalysis. The magnetic character of the catalyst and support will allow easy recovery and reuse during the catalytic Fenton reaction.

#### 3.1.3. FTIR Analysis

**Figure 3:**
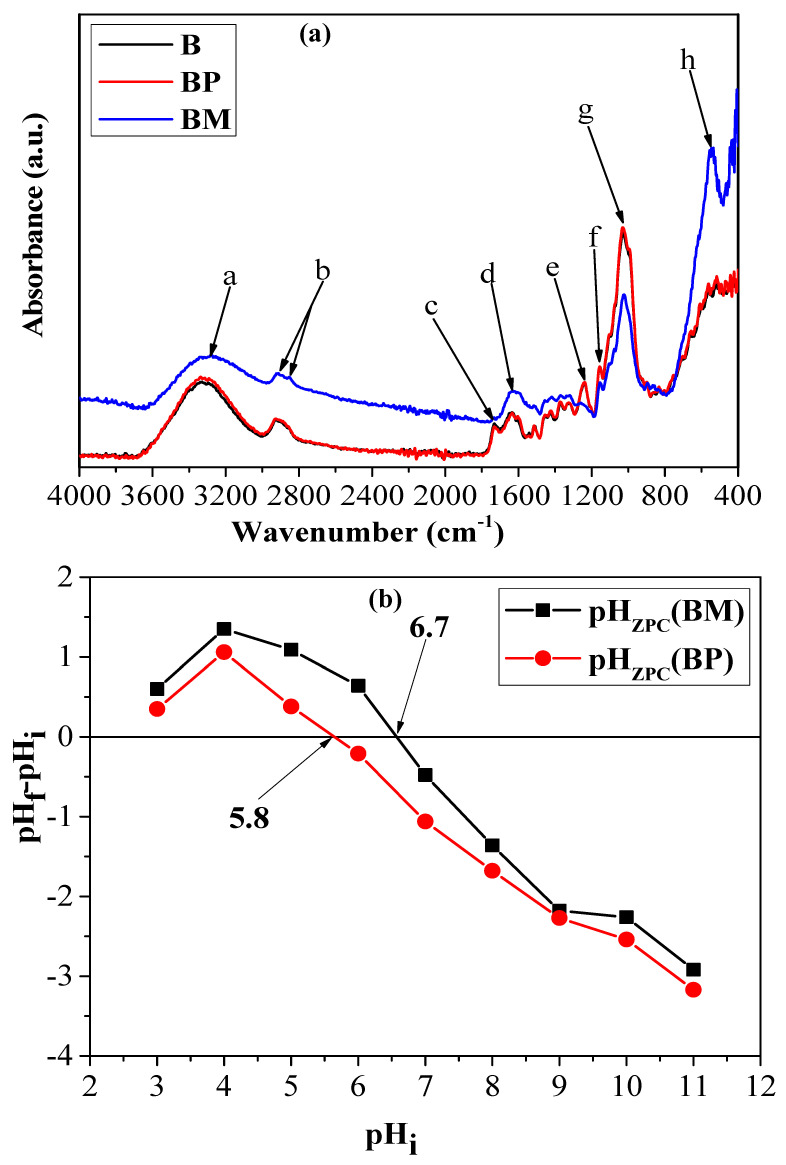
(**a**) FTIR spectra of bagasse fibers before (B) and after (BP) plasma-activated, and supported magnetite (BM); (**b**) zero-point charge (pH_ZPC_) of plasma-modified bagasse (BP) and supported magnetite (BM).

The FTIR spectra (chemical functions on the surface) of raw bagasse fibers, plasma-activated bagasse and supported magnetite were recorded, as we can see in Figure 3a and Table 1 with assignment. The results show the presence of chemical bonds of lignin, hemicellulose and cellulose in the sugarcane bagasse fibers (B). The plasma treatment induces a slight intensity increase in the absorption band at approximately 3336 cm^−1^ ascribed to the stretching vibration of the HO- group of carboxylic acids, phenols, cellulose and hemicellulose due to the grafting of hydroxyl groups (HO-) on the unsaturated bonds [21,27]. The low intensity band at approximately 2925 cm^−1^ corresponds to the symmetric and asymmetric stretching vibrations of the C-H group of alkyls in the aliphatic chains of cellulose, lignin and hemicellulose. The absorption band at approximately 1732 cm^−1^ is attributed to the bending vibration of C=O groups present in the lignin [21]. The band at approximately 1650 cm^−1^ represents the absorbed water from the cellulose and can vary following the nature of the sample [42], and the band between 1250 and 1400 cm^−1^ reflects the vibrations of the aromatic C=C group of lignin. The bands at 1250 and 1000 cm^−1^ are assigned to the stretching vibrations of C-O groups within the alcohols, phenols or ether components of the lignocellulosic structure of sugarcane bagasse fibers [42]. The vibration band at approximately 565 cm^−1^ is attributed to the Fe-O bond of magnetite (Fe_3_O_4_), which once confirms their incorporation within the bagasse matrix [43]. The results corroborate those of Miloh et al. (2024) [27], reporting the fact that even if the magnetite nanoparticles are well incorporated within the organic matrix of hyacinth biomass used as support, they do not cover the entire surface like a core-shell. This suggests that the active sites generated by plasma serve as anchoring sites for magnetite particles.

Figure 3b depicts the zero-point charge (pH_ZPC_) of plasma-activated bagasse fibers (BP) and supported magnetite (BM) with the respective values of 5.8 and 6.7. Therefore, the surface of both materials will be positively charged for pH values lower than their respective pH_ZPC_ values [27], favoring a better adsorption of the negative charges of AR molecules. However, their surface will be negatively charged for pH values of the solution higher than their respective pH_ZPC_ values, inducing a repulsion with the negative charges of AR molecules and inhibiting the adsorption and catalytic processes.

### 3.2. Degradation of AR by the Heterogeneous Fenton Process in Plasma Medium

#### 3.2.1. Parameters’ Optimization by Plasma Alone

**Figure 4:**
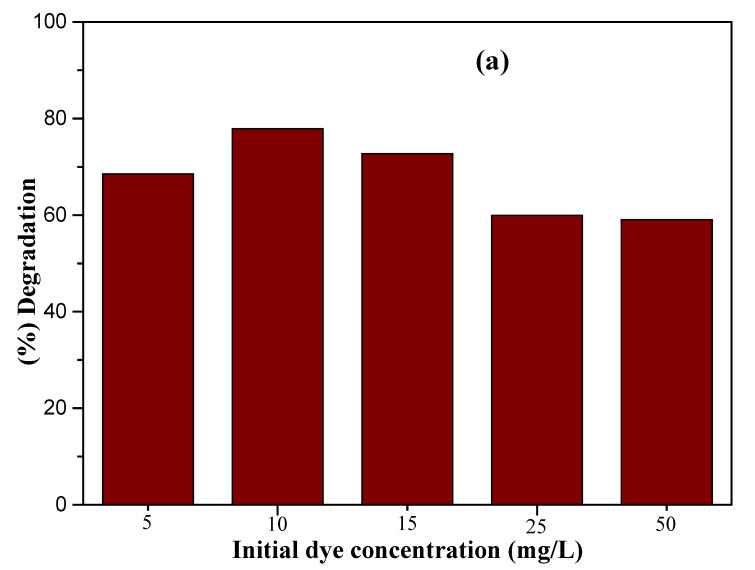

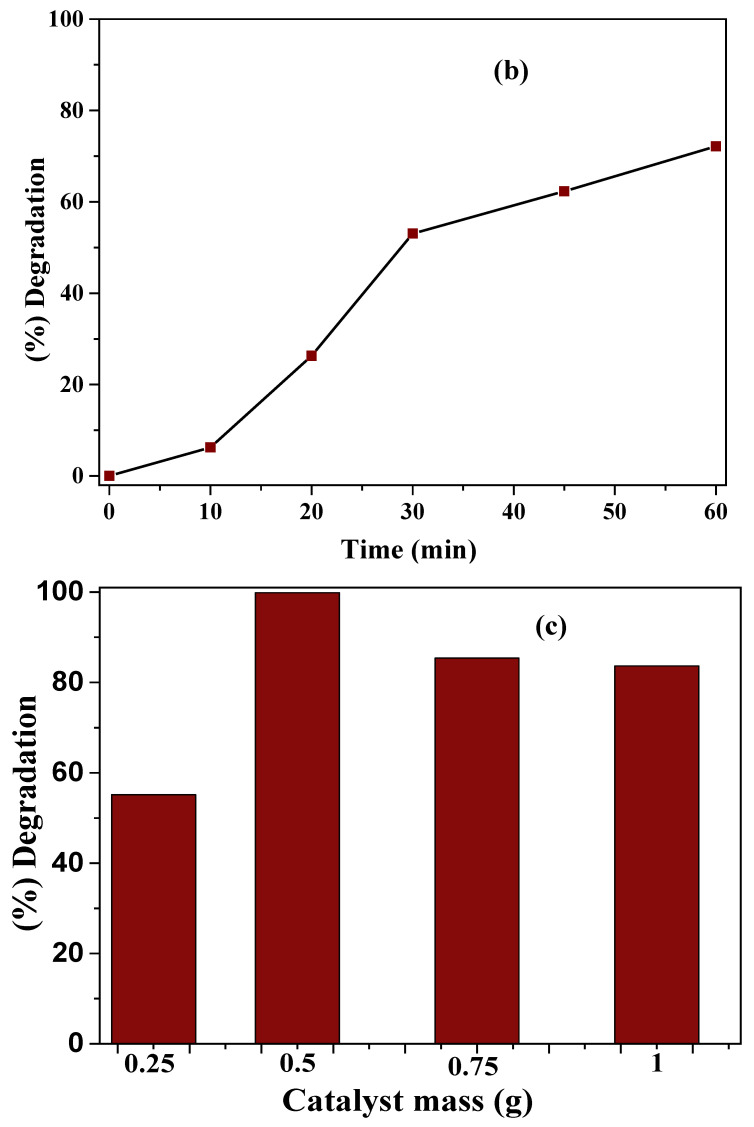

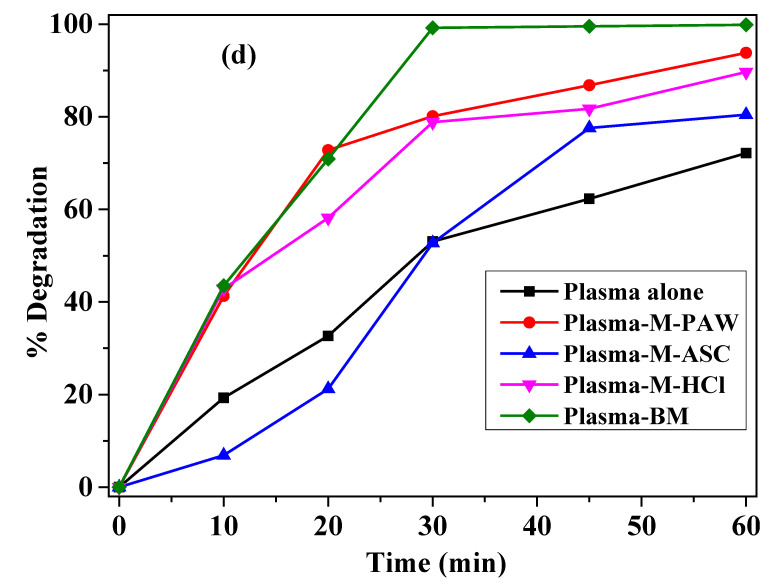
Influence of AR concentration (**a**) and exposure treatment time (**b**) on the degradation rate of AR by plasma alone; (**c**) influence of the catalyst mass; (**d**) study of the degradation of AR by heterogeneous plasma/Fenton coupling using prepared catalysts.

Some experimental parameters are likely to influence the degradation process of pollutants during the Fenton heterogeneous catalysis process in a plasma medium. The influence of the pollutant initial concentration was studied by using AR solution concentration values of 5, 10, 15, 25 and 50 mg/L at pH = 5.7, placed in contact with the electric discharge for 30 min and the results are shown in Figure 4a. It is observed in Figure 4a that when the initial dye concentration increases up to 10 mg/L, the reactivity between the pollutant molecules and the radical species generated by the plasma also increases, thus contributing to the improvement of the degradation efficiency. It appears that the increase in the initial concentration of AR dye promotes the possibility of interaction between the target molecules of the dye and the HO^•^ plasma generated. Meanwhile, when the concentrations of pollutant molecules are higher than 10 mg/L (78% reached), the degradation efficiency gradually decreases, and a degradation rate of 59.1% is obtained for a concentration of 50 mg/L. This trend can be attributed to the fact that at lower concentrations (10 mg/L), there are enough plasma-generated oxidizing species that enable them to interact with the dye molecules, thus allowing more efficient degradation. As the concentration increases, plasma-generated oxidizing species are insufficient, limiting the degradation efficiency [46,47]. Previous work has shown that for high concentrations of pollutant molecules, the generation of radicals (HO^•^, NO^•^) useful for the rupture of the dye chromophores is insufficient [34]. Thus, the degradation efficiency is less good for high concentrations of pollutant molecules using plasma alone (without catalyst). For the optimal pollutant concentration of 10 mg/L, the variation in treatment time shows, according to Figure 4b, an increase in the degradation rate with the treatment time. A quasi-stabilization of the degradation rate is recorded between 30 and 60 min, which could reflect a balance between the species produced by the plasma and those already available in solution. Therefore, 10 mg/L and 30 min appear, respectively, as optimal dye concentration and exposure time.

#### 3.2.2. Study of the Degradation of AR by the Heterogeneous Fenton Process Coupled with Plasma

To increase the degradation rate obtained by plasma alone (without catalyst), a study of AR dye degradation was carried out by coupling the plasma route with the obtained bulk and supported magnetite as catalysts. First, the optimal mass of the catalyst was found by varying the mass of supported magnetite material (BM). As we can see in Figure 4c, the degradation efficiency increases until 0.5 g and decreases above 0.5 g due to the huge presence of catalyst, inhibiting the penetration of the plasma UV light within the solution, thus inhibiting the photocatalytic process at the catalyst’s surface. Therefore, 0.5 g appears as the optimal catalyst mass and will be used for the continuation of the study. Figure 4d shows the evolution of degradation rate during plasma-coupling with the four prepared catalysts (M-PAW, M-HCl, M-ASC and BM). The heterogeneous plasma/Fenton coupling provides much better results compared to the plasma treatment alone, with the degradation rate increasing from 53.06% (plasma treatment alone) to 80.13% (plasma coupling + M-PAW), 78.85% (plasma coupling + M-HCl) and 99.24% (plasma coupling + BM) after 30 min. The analysis of the chemical composition showed the presence of iron in different oxidation states, Fe(II) being a reagent of the Fenton reaction. H_2_O_2_ generated in post-discharge plasma glidarc, as secondary species, induces the Fenton catalytic reaction, given by Equations (7)–(9), thus producing more HO^•^ radicals responsible for the degradation of organic molecules. Moreover, plasma UV illuminates the catalyst’s surface (photocatalytic route), producing another HO^•^ and HO_2_^•^ radicals via the redox reactions between the electrons (conduction band) and holes (valence band) [48], respectively, with dissolved oxygen and water in solution. Therefore, a synergistic effect between the plasma route and Fenton reaction generates a large quantity of HO^•^ radicals and the regeneration of the Fenton reagent, which contributes to increasing the degradation rates [49]. It is also noted that the degradation rates during the different couplings depend on the nature of the antioxidant agent used. Scanning electron microscopy images (Figure 2) have shown that the shape of the iron oxide particles was a function of the iron oxidation inhibitor agent used. The catalytic degradation of AR, which is preceded by the adsorption of pollutant molecules on the surface of the catalyst [50], depends strongly on the topography of the materials. Following the efficiency order, we have the following: BM > M-PAW > M-HCl > M-ASC, i.e., nanorods are more efficient than nanospheres, which are more efficient than nanosheets. Equation (9) generally translates the mechanism of plasma chemical degradation of pollutants. Table 2 summarizes a comparative study of the catalytic degradation of AR dye using the prepared materials in the present work, compared to the literature data, and reveals the effectiveness of the prepared magnetite following the exposure time, dye and catalyst dose.

Fe^2+^ + H_2_O_2_ ⇌ Fe^3+^ + HO^•^ + HO^−^ (7)

Fe^3+^ + H_2_O_2_ ⇌ Fe^2+^ + HOO^•^ + H^+^ (8)

HO^•^ + pollutants + O_2_ → CO_2_+ H_2_O + intermediate products (9)

**Figure 5:**
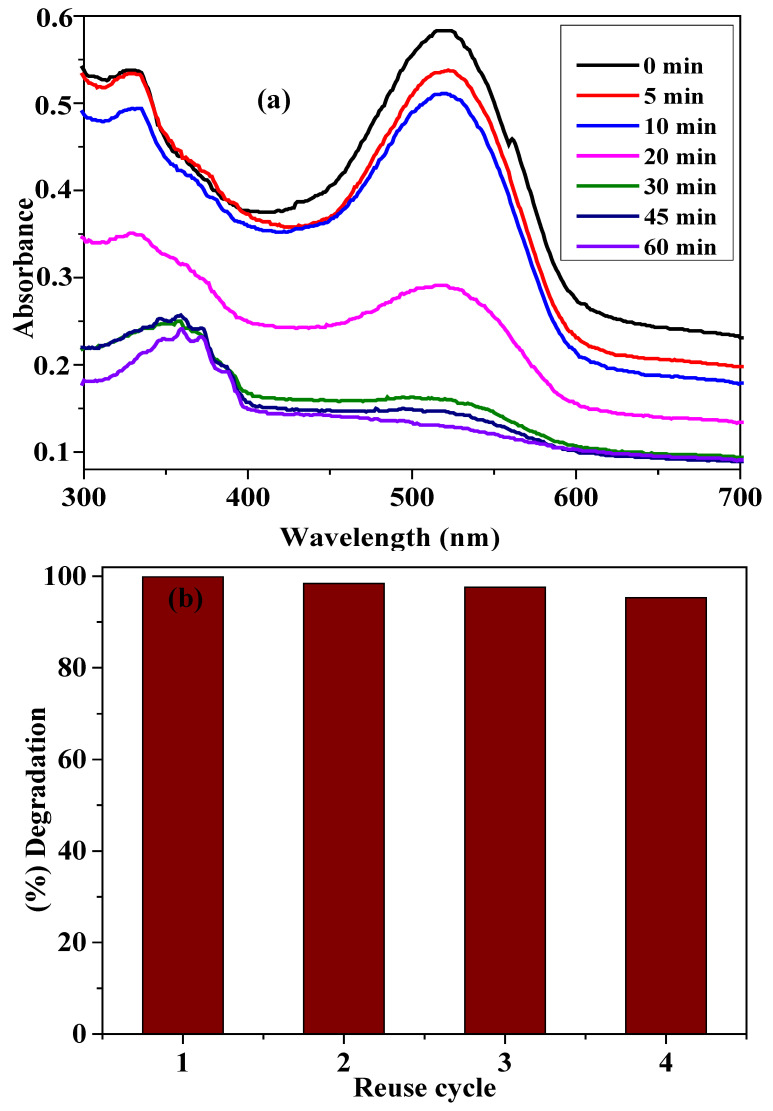
(**a**) UV–vis spectra evolution of AR dye with BM catalyst; (**b**) recyclability test of supported magnetite (BM) during plasmacatalytic degradation of AR dye.

To further attest to the degradation process of the AR dye molecule after adsorption at the catalyst’s surface, the UV–vis spectra evolution has been recorded at defined treatment times. As we can see in Figure 5a, the intensity peak at approximately 520 nm, ascribed to the chromophore’s groups (-N=N-), which is responsible for the AR dye coloration [34], significantly decreases after 30 min of treatment, thus suggesting the destruction of the chromophores’ groups. Therefore, the improvement of the plasma treatment alone (53.06%) is achieved by coupling with the BM catalyst (99.24%). This can be attributed to the fact that, in addition to the hydroxyl radical (HO^•^) as a powerful oxidizing species, the heterogeneous Fenton reaction through supported magnetite and H_2_O_2_ plasma-generated as a secondary species also produces additional HO^•^. Thus, a synergetic effect between plasma and catalyst leads to a significant degradation efficiency of the complex structure of AR dye via chromophore groups and -C=C- of the aromatic cycle (naphthalene) [44]. A similar observation was made by Salem et al. (2016) during the removal of amaranth red dye using Fe_3_O_4_/MgO catalyst [51]. The catalytic stability (reusability) of the prepared magnetite biocomposite (BM) based on the plasma-activated bagasse sugarcane fibers as support has been performed during four consecutive catalytic cycles, and the results obtained are shown in Figure 5b. We observe that the degradation efficiency of BM catalyst remains almost stable during the four plasmacatalytic cycles with respective values of 100, 99, 98 and 96%, thus highlighting, on the one hand, the useful and benefit of supporting magnetite particles on the plasma-activated sugarcane bagasse fibers, and on the other hand, the ferromagnetic magnetic test (Figure S4), allowing an easy catalyst recovery. Our supported magnetite on the plasma-activated sugarcane bagasse fibers reveals a higher catalytic stability compared to Fe_3_O_4_/MgO prepared by Salem et al. (2016) using the sol-gel method, which loses amaranth red (AR) removal efficiency (about 20%) after the fourth cycle [51]. In our previous study (Miloh et al. 2024), the supported magnetite on the plasma-activated hyacinth biomass reveals the pollutant degradation rates of 97, 96, 92 and 85%, respectively, after four catalytic cycles [27]; thus, the decrease in efficiency is probably due to the detachment of magnetite particles (active phase) from the hyacinth biomass (support). Therefore, the present work highlights a better deposition of magnetite particles on the bagasse sugarcane fibers through strong chemical bonds (in the present work) compared to hyacinth biomass used in previous work [27], which could be explained by the fact that the surface energy of plasma-activated bagasse sugarcane is higher than the plasma-activated hyacinth.

**Table 2:** Comparison study of degradation efficiency of AR dye using bulk and supported magnetite with the literature data.

Catalyst	[AR] (mg/L)	[Catalyst] (g/L)	Time (min)	Degradation Rate (%)	Irradiation Source	Reference
MnO_2_	50	1.0	30	47	Plasma UV light	[34]
MnFe_2_O_4_@ZnO	20	Not reported	240	60	UV–Vis light	[52]
ZnO/C	10	Not reported	480	86	UV lamp	[53]
β-Ga_2_O_3_	20	0.04	165	97	UV lamp	[54]
C_3_N_4_BiOI supported	10	1	150	84	Visible light	[55]
M-PAW	10	1.16	30	80	Plasma UV light	Current work
M-HCl	10	1.16	30	79	Plasma UV light	Current work
M-ASc	10	1.16	30	53	Plasma UV light	Current work
BM	10	1.16	30	99	Plasma UV light	Current work
BM	50	1.16	30	24	Plasma UV light	Current work

#### 3.2.3. Kinetic Monitoring of Plasmacatalytic Degradation of AR Dye

**Figure 6:**
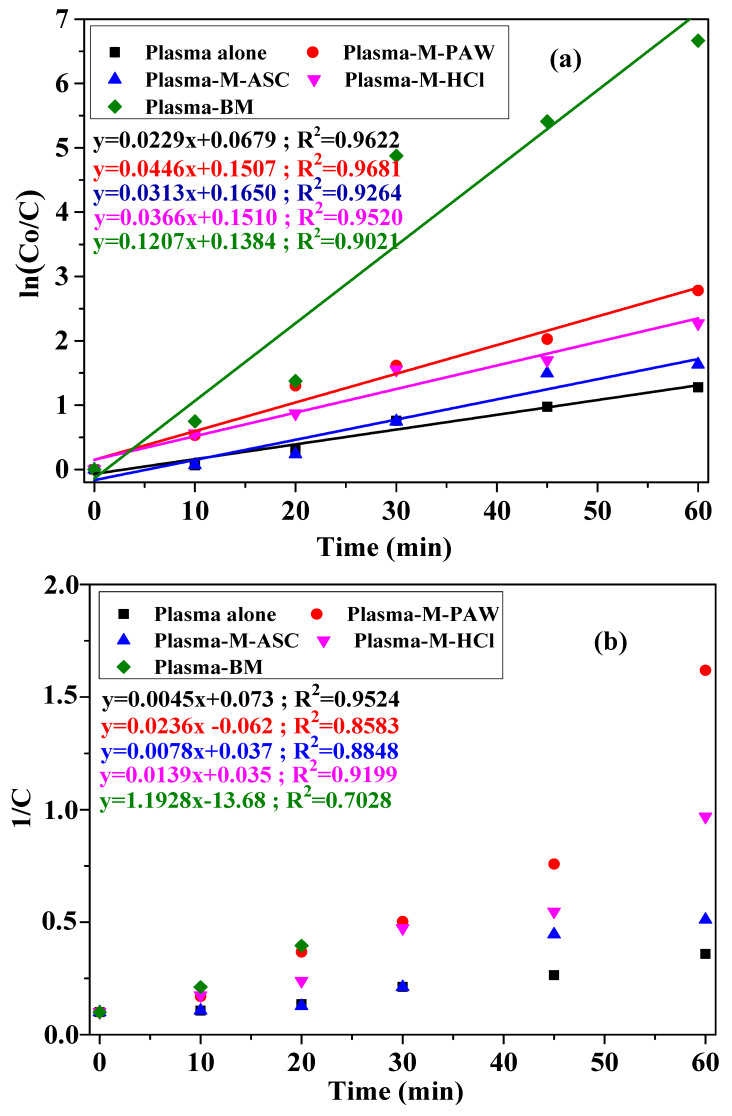

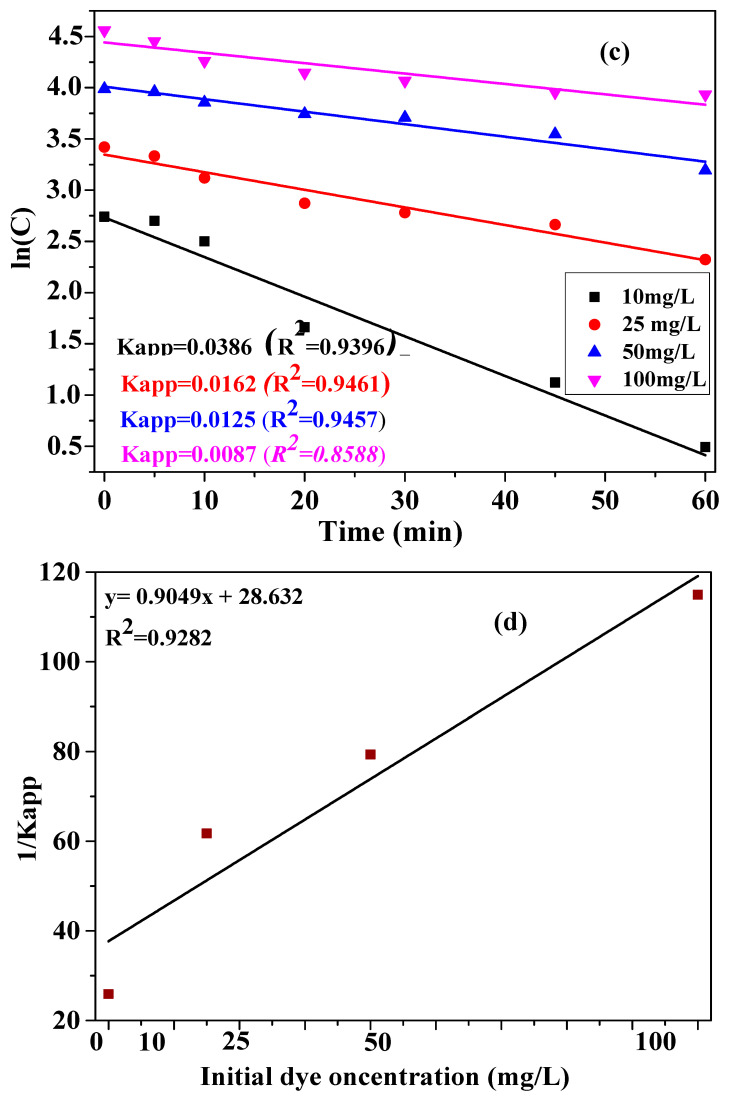
Study of the first (**a**) and second (**b**) order kinetics during degradation of AR dye; (**c**,**d**) Langmuir–Hinshelwood kinetics study using BM material.

The 1st order {ln(C_o_/C) = f(t)} and 2nd order {1/C = f(t)} kinetic models were plotted for all materials used during the plasma-catalyst coupling. Figure 6 and Table 3 provide the values of the regression coefficients obtained according to each kinetic model. The plotting of the first (Figure 6a) and second (Figure 6b) order kinetic models clearly shows that the first-order kinetic model better describes the experimental data because all the regression coefficients (R^2^) obtained are close to unity and are higher than those obtained by the second pseudo-second-order model (Table 3). The Fenton plasmacatalytic degradation of AR pollutant is better described by the pseudo-first-order kinetic model, following the regression coefficient value (R^2^) close to the unit, thus illustrating the fact that the degradation is controlled by the generated species in solution during the treatment. Therefore, the adsorption of pollutant molecules on the surface of the materials is the limiting step of the AR dye degradation process by the heterogeneous Fenton process in plasma medium using magnetic iron oxides [56]. This observation invites us to plot the Langmuir–Hinshelwood kinetic model to determine the adsorption equilibrium constant and the reaction rate constant.

#### 3.2.4. Langmuir–Hinshelwood Kinetic Model

The BM material that provided the best results during the plasma-catalyst coupling was used for the Langmuir–Hinshelwood kinetics. The plot of the logarithm of the residual concentration as a function of the treatment time allowed the finding of the apparent reaction constants for the three concentrations used for this experiment (Figure 6c). The plot of Figure 6d with a regression coefficient close to unity allowed the finding of the parameters K_LH_ and K_r_ from the slope (1/Kr) and the intercept (1/K_r_*K_LH_). The adsorption equilibrium constant (K_LH_) and reaction rate constant (K_r_) are, respectively, 0.0316 L.mg ^−1^ and 1.1051 mg.L^−1^.min^−1^. It is observed that during the first 10 min of plasmacatalytic treatment, the concentrations of AR decrease slightly, and the pseudo-equilibrium recorded could be ascribed to the desorption process of the pollutant molecules (AR) from the BM composite surface after the adsorption step. Table 4 provides the values of the apparent reaction constants for concentrations of 10, 25, 50 to 100 mg/L. An increase in the reaction constant is observed with increasing concentration, which implies that the diffusion of AR pollutant molecules controls their degradation by radical species produced concomitantly by the plasma route and Fenton reaction [47].

## 4. Conclusions

The antioxidant agents used in this work influence the morphology of bulk and supported magnetite nanoparticles prepared by the co-precipitation route. Nanorods of magnetite were obtained with plasma-activated water, nanospheres with hydrochloric acid (HCl), nanosheets with ascorbic acid (ASC) and nanospheres dispersed on the sheet for supported material (BM) using PAW as an antioxidant agent. Physicochemical analyses highlighted the deposition of magnetite (Fe_3_O_4_) on the plasma-activated bagasse sugarcane fibers. The heterogeneous plasma/Fenton coupling revealed a higher removal efficiency of the AR pollutant compared to the plasma treatment alone. Supported magnetite (BM) based on the plasma-activated bagasse sugarcane fibers revealed significant catalytic stability during the four cycles, thus highlighting the easy recovery and reuse. Plasma-activated water (PAW) and bagasse sugarcane fibers appear, respectively, to be the best and cheapest oxidation inhibitor agent of Fe(II) to Fe(III) and support for the magnetite composite synthesis. The study of the pseudo-first and -second-order kinetics models highlights the fact that the Fenton plasma-catalytic degradation of the AR dye molecule using the prepared BM composite is better described by the pseudo-first-order kinetic model. This work highlights the multifunctionality of gliding arc plasma route as a device to (i) produce the plasma-activated water (PAW) used as a good oxidation inhibitor for synthesis of bulk magnetite nanomaterials via co-precipitation route, (ii) activate the surface of bagasse sugarcane fibers as a catalytic support (iii) production of H_2_O_2_ species for Fenton catalytic route in addition to UV light for photocatalytic route, and primaries oxidizing species (HO^•^ and HO_2_^•^) generated, for an effective degradation of the organic pollutant in solution.

## Figures and Tables

**Table 1:** IR absorption of bagasse fibers before (B) and after (BP) plasma-activated, and supported magnetite (BM).

Wavelength Range (cm^−1^)	B	BP	BM	Corresponding Chemical Function	Reference
(a) 3600–3200	3329	3336	3279	Stretching vibration of O-H of cellulose and hemicellulose	[21,27,44]
(b) 3000–2800	2923	2925	2912	Symmetric and asymmetric vibrations of C-H from aliphatic group –CH_2_ of cellulose and lignin	[21,27,41]
(c) 1745–1730	1732	1732	/	Bending vibration of carbonyl groups C=O present in the lignin	[21]
(d) 1650–1630	1634	1635	1627	Bending vibration of OH from adsorbed water into the cellulose	[42]
(e) 1250–1400	1237	1242	1320	Vibration of aromatic C=C bonds of lignin	[41]
(f) 1156	1156	1155	1154	Asymmetric stretching of C-O-C bond	[45]
(g) 1000–1250	1030	1031	1022	Asymmetric bending vibration of C-O group into hemicellulose and lignin	[46]
(h) 500–700	/	/	567	Vibration Fe–O bond	[47]

**Table 3:** Correlation coefficient data of the first- and second-order kinetics.

Treatment	First Order (R^2^)	Second Order (R^2^)
**Plasma alone**	0.9622	0.9524
**Plasma + M-PAW**	0.9681	0.8583
**Plasma + M-ASC**	0.9264	0.8848
**Plasma + M-HCl**	0.9520	0.9199
**Plasma + BM**	0.9021	0.7028

**Table 4:** Langmuir–Hinshelwood kinetics study: Apparent reaction constants.

Materials	Concentration (mg/L)	Kapp (min^−1^)	R^2^
**Plasma-BM**	10	0.0386	0.9396
	25	0.0162	0.9461
	50	0.0125	0.9457
	100	0.0087	0.8588

## Data Availability

The original contributions presented in this study are included in the article/Supplementary Materials. Further inquiries can be directed to the corresponding authors.

## Supplementary Materials

The following supporting information can be downloaded at: https://www.mdpi.com/article/10.3390/polym18141730/s1. Figure S1: Experimental scheme of the Glidarc plasma device; Figure S2: Molecular structure of Amaranth Red dye.; Figure S3: EDX spectra of (a) M-PAW, (b) M-HCl, (c) M-ASC, (d) BP and (e) BM materials; Figure S4: Picture of the Magnet test of synthesized magnetite.
