# Supplementary material for: Magnetite (Fe3O4) Supported on Bagasse Sugarcane Fibers as Catalyst for Plasma-Degradation of Organic Pollutant in Water: Effect of Oxidation Inhibitor Agents on the Particles’ Shape and Catalytic Activity
Source: Polymers (Basel). 2026 Jul 14;18(14):1730. doi: 10.3390/polym18141730 (PMC13417173; doi:10.3390/polym18141730)
Supplement: Supplementary file 1 [file polymers-18-01730-s001.zip › polymers-4045032-supplementary.pdf]

Magnetite (Fe_3O_4) Supported on Bagasse Sugarcane Fibers as Catalyst for Plasma-Degradation of Organic Pollutant in Water: Effect of Oxidation Inhibitor Agents on the particles Shape and Catalytic Activity.

Néhémie Miloh, Franck W. Boyom-Tatchemo*, Fabrice Nganbe-Ndjock, Albert B. Mbouopda-Poupi, Elie Acayanka*, Georges Kamgang-Youbi

University of Yaounde I, Faculty of Science, Department of Inorganic Chemistry, Cameroon

Corresponding authors: boyomtatchemofranckwilliam@gmail.com (F.W. Boyom-Tatchemo), acayanka@gmail.com (E. Acayanka).

Supplementary files

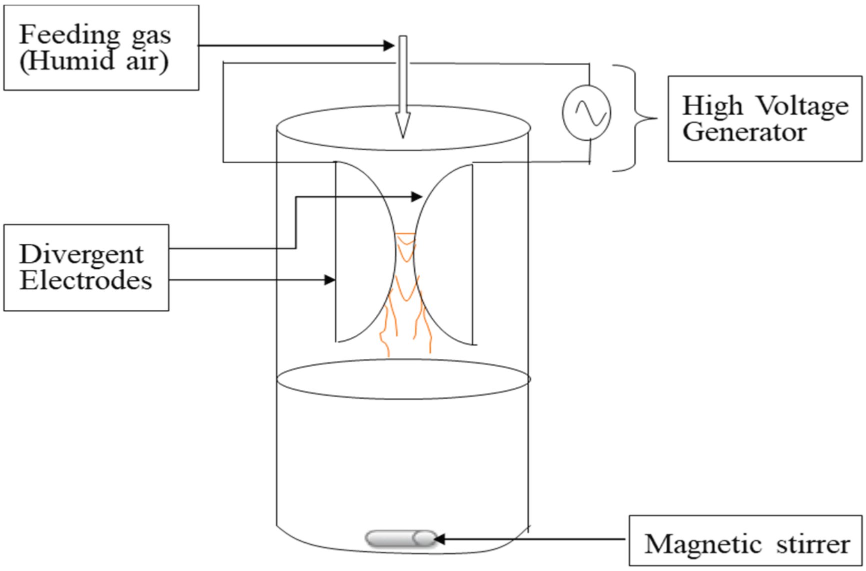

Figure S1: Experimental scheme of the Glidarc plasma device.

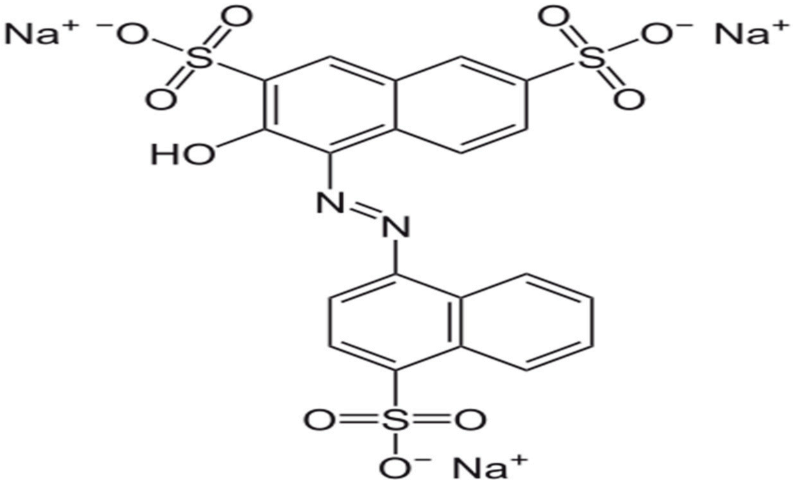

Figure S2: Molecular structure of Amaranth Red dye.

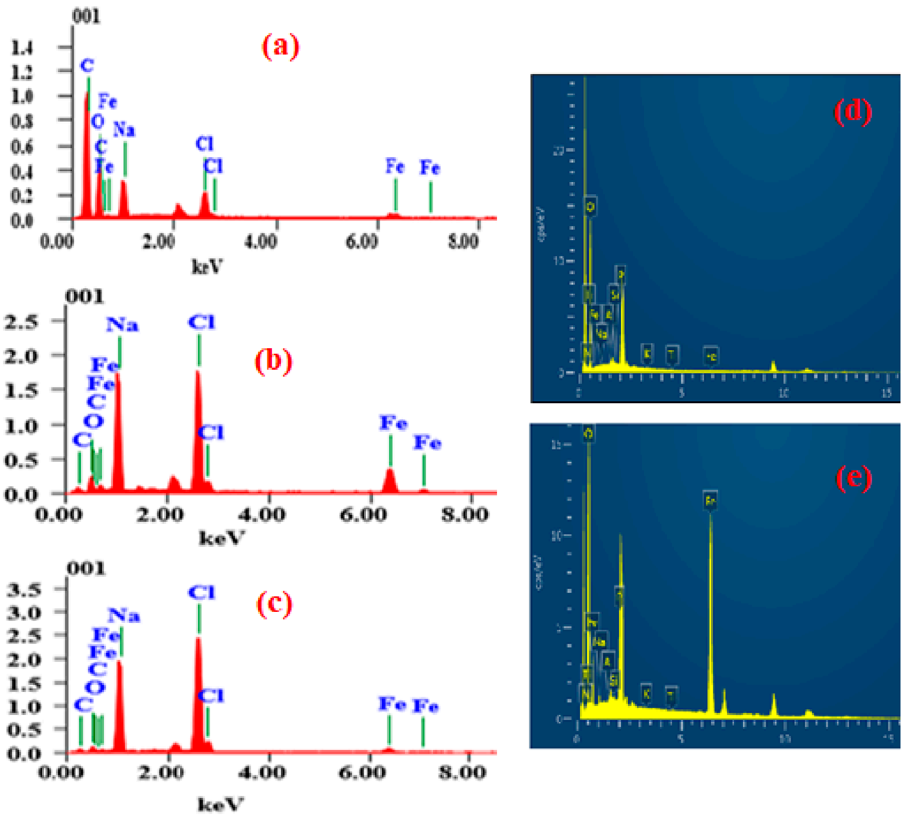

Figure S3: EDX spectra of (a) M-PAW, (b) M-HCl, (c) M-ASC, (d) BP and (e) BM materials.

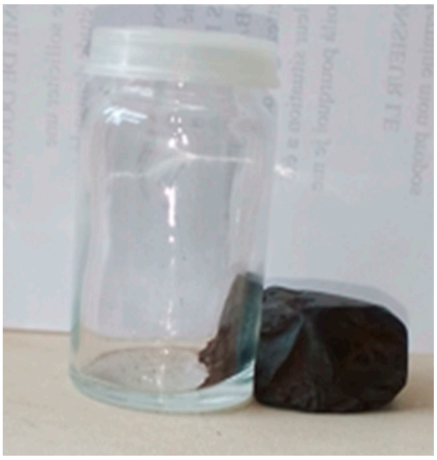

Figure S4: Picture of the Magnet test of synthesized magnetite.

--
